# P-790. Cephalexin Versus Cefdinir for the Treatment of Uncomplicated Urinary Tract Infections

**DOI:** 10.1093/ofid/ofaf695.1001

**Published:** 2026-01-11

**Authors:** Tyler Mitzner, Lisa E Dumkow, Kristen Eid, Destiny Hughson, Andrew Jameson

**Affiliations:** Trinity Health Grand Rapids, Inverness, IL; Trinity Health Grand Rapids, Inverness, IL; Trinity Health Grand Rapids, Inverness, IL; Trinity Health Grand Rapids, Inverness, IL; Trinity Health Grand Rapids, Inverness, IL

## Abstract

**Background:**

Due to its low bioavailability and poor urinary penetration, there are concerns that cefdinir is a suboptimal agent for the treatment of urinary tract infections (UTI). There is limited available literature examining cefdinir’s use in UTIs which has not shown worse outcomes compared to other oral beta-lactams; however, these studies had significant limitations, such as small sample size, short periods of follow up, or lack of assessment of clinical cure.

**Methods:**

This retrospective, multicenter cohort study included adult female patients who received either cephalexin or cefdinir for 5 to 7 days for symptomatic uncomplicated UTI (uUTI). The primary objective was to compare uUTI treatment failure, defined as recurrent or continued symptoms within 30 days, between patients treated with cephalexin 500 mg twice daily (cephalexin group) and cefdinir 300 mg twice daily (cefdinir group) in the outpatient setting. Secondary outcomes included time to treatment failure, adverse events within 7 days of treatment, and *Clostridioides difficile* within 30 days of treatment.

**Results:**

A total of 367 patients were included (cephalexin, n = 200; cefdinir, n = 167). Patients in both groups were treated for a median of 7 days. Patients treated with cefdinir experienced a significantly higher rate of treatment failure (cephalexin 12.5% vs. cefdinir 23.4%, p=0.006), and cefdinir was independently associated with treatment failure (OR: 1.9 [95% CI: 1.1-3.4]). Additionally, patients who experienced treatment failure with cefdinir had a higher incidence of cefazolin-non-susceptible pathogens (cephalexin 0% vs. cefdinir 37.5%; p=0.024) and ceftriaxone-non-susceptible pathogens (cephalexin 0% vs. 31.2%; p=0.053) on repeat culture.

**Conclusion:**

Cefdinir was independently associated with treatment failure with nearly twice as high of failure rate compared with cephalexin for the treatment of outpatient uUTI. Patients who failed treatment with cefdinir were more likely to demonstrate first- and third-generation cephalosporin resistance on subsequent urine culture.

**Disclosures:**

All Authors: No reported disclosures

